# Reduced specificity for the local lymph node assay for lipophilic chemicals: Implications for the validation of new approach methods for skin sensitization

**DOI:** 10.1016/j.yrtph.2023.105333

**Published:** 2023-02

**Authors:** Andreas Natsch, Nicole Kleinstreuer, David Asturiol

**Affiliations:** a Fragrances S&T, Ingredients Research, Givaudan Schweiz AG, Kemptthal, Switzerland; b National Toxicology Program Interagency Center for the Evaluation of Alternative Toxicological Methods, RTP, NC, USA; c European Commission, Joint Research Centre (JRC), Ispra, Italy

**Keywords:** Skin sensitization, Local lymph node assay, Alternative assays, Lipophilic chemicals, Predictivity

## Abstract

Meaningful and accurate reference data are crucial for the validation of New Approach Methodologies (NAMs) in toxicology. For skin sensitization, multiple reference datasets are available including human patch test data, guinea pig data and data from the mouse local lymph node assay (LLNA). When assessed against the LLNA, a reduced sensitivity has been reported for *in vitro* and *in chemico* assays for lipophilic chemicals with a LogP ≥3.5, resulting in reliability restrictions within the h-CLAT OECD test guideline.

Here we address the question of whether LLNA data are an appropriate reference for chemicals in this physicochemical range. Analysis of LLNA vs human reference data indicates that the false-discovery rate of the LLNA is significantly higher for chemicals with LogP ≥3.5. We present a mechanistic hypothesis whereby irritation caused by testing lipophilic chemicals at high test doses leads to unspecific cell proliferation. The accompanying analysis indicates that for lipophilic chemicals with negative calls in *in vitro* and *in chemico* assays, resorting to the LLNA is not necessarily a better option. These results indicate that the validation of NAMs in this particular LogP range should be based on a more holistic evaluation of the reference data and not solely upon LLNA data.

## Highlights

• Analysis of the predictivity of *in vitro* methods against a highly curated LLNA database.
• Higher False Detection Rate of the LLNA for lipophilic chemicals.
• Weight of evidence analysis to understand potential false-positive chemicals in the LLNA.
• Validation of NAMs for lipophilic chemicals should not be based solely upon LLNA data.
• Irritation caused by testing lipophilic chemicals at high test doses leads to unspecific cell proliferation.

## Abbreviations:

NAM: New Approach Methodologies
h-CLAT: human cell line activation test
LLNA: local lymph node assay
DMSO: dimethyl sulfoxide;
OECD: Organization for Economic Cooperation and Development
AOP: adverse outcome pathway
DPRA: direct peptide reactivity assay
TG: test guidelines
ARE: antioxidant response element
ICATM: International Cooperation on Alternative Test Methods
GHS: global harmonized system
DA: defined approaches
DASS: DAs for skin sensitization
WNT: working group of national coordinators of the test guidelines programme
HDSG: human data sub-group;
NICEATM: National Toxicology Program Interagency Center for the Evaluation of Alternative Toxicological Meth-ods
HRIPT: human repeat insult patch tests
HMT: human maximisation tests
NC: negative call
FDR: False-discovery rate
FN: False-negative
FP: False-positive
TN: True-negative
TP: True-positive
DASS DB: database of the DASS group
WoE: weight of evidence
GPMT: guinea pig maximisation test
SDS: sodium dodecyl sulphate
ICCVAM: Interagency Coordinating Committee on the Validation of Alternative Methods

## 1 Introduction

Allergic contact dermatitis, or skin sensitization, is a prevalent occupational and public health concern. In 2013, the Organization for Economic Cooperation and Development (OECD) published an adverse outcome pathway (AOP) for skin sensitization linking molecular initiating events and cellular and tissue effects in the sensitization process (OECD, 2014). Since then, several New Approach Methodologies (NAMs) covering *in vitro* (cell-based) and in chemico (cell-free) testing methods for assessment of skin sensitization mapped to key events in the AOP have been validated in international interlaboratory ring-trials. Currently, there are OECD test guidelines (TG) based on four key events of the AOP: (1) Covalent binding with skin proteins (haptenation) measured by in chemico assays, e.g. the Direct peptide reactivity assay (DPRA) TG 442C; (OECD, 2020); (2) activation of the Keap1/Nrf2 pathway by electrophilic substances, as monitored by the antioxidant response element (ARE) assay, e.g. KeratinoSens™ in TG 442D (OECD, 2018a); (3) dendritic cell activation, e.g. via the human cell line activation test (h-CLAT) in TG 442E (OECD, 2018b); and (4) lymphocyte proliferation, as measured by the *in vivo* murine local lymph node assay (LLNA) in TG 429 (OECD, 2010). Among the animal tests, the LLNA is currently the regulatory standard for assessing skin sensitization potential, although the guinea pig test method (OECD, 2022) is still requested by some regulatory authorities under specific circumstances (e.g. for certain medical device products).

At this stage, none of the individual NAMs are accepted as stand-alone replacements for the LLNA, but combinations of these methods (so-called “defined approaches” or DAs) have been developed and were submitted to the OECD as case studies (OECD, 2016). These DAs providing chemical hazard and potency predictions range from simple rule-based decision trees to more complex machine learning algorithms that represent nonlinear combinations of *in vitro*/in chemico NAM data, chemical structural features and physicochemical properties. Work performed in collaboration with the Cosmetics Europe industry consortium demonstrated that many of the DAs provide superior performance to the existing animal tests when compared to human data (Kleinstreuer et al., 2018). Beginning in 2017, an OECD project led by the US, Canada, and the EU was established to curate high-quality reference datasets of unprecedented size and to develop a novel internationally harmonized DA guideline to predict chemical skin sensitization potential. The International Cooperation on Alternative Test Methods (ICATM) used the DAs for skin sensitization as a case study to develop an assessment framework for establishing confidence in DAs (Casati et al., 2018), and this framework was used to evaluate multiple DAs for inclusion in the guideline. The OECD TG 497 on DAs for skin sensitization (DASS), the first phase of which covers simple rule-based DAs, was adopted in 2021 (OECD, 2021) as the first of its kind and provides full replacement alternatives to the existing *in vivo* methods. The validated DAs contained in the OECD guideline were extensively characterized and shown to either provide the same level of information or be more informative than the LLNA (OECD TG 429) for hazard identification (i.e. sensitizer versus non-sensitizer) and for GHS potency categorization. The DASS Guideline includes workflows for identifying borderline results, characterizing the applicability domain of the DAs, and assigning confidence to the predictions.

Originally, the objective of the DAs was to overcome the limitations of the individual information sources, and the overall performance analysis against the reference datasets (LLNA and human) supported this lack of restrictions. However, the OECD working group of national coordinators of the test guidelines programme (WNT) supported a more conservative approach whereby the limitations of the individual test methods, as defined by their respective TGs, were carried forward and applied to the DAs that use them. For example, a reduced sensitivity of the h-CLAT as a stand-alone method vs LLNA data for chemicals with a LogP ≥3.5 had been reported (Takenouchi et al., 2013) and was included as a limitation in OECD TG 442E (OECD, 2018b). This limitation was therefore automatically applied to the DAs that incorporate the h-CLAT as an information source. While analysing predictivity of the *in vitro* tests as stand-alone methods vs LLNA data based on the curated DASS reference database, a reduced sensitivity of all three tests used as DASS information sources, i.e. OECD TG 442C (DPRA), 442D (KeratinoSens™) and 442E (h-CLAT), for chemicals with a LogP ≥3.5 was also observed. However, the potential for false-positives in the LLNA test has been previously noted, and was a significant point of discussion both during and after the LLNA validation (Ball et al., 2011; Basketter and Kimber, 2011; Kreiling et al., 2008; Ku et al., 2008; Montelius et al., 1994). In fact, there was a limited number of non-sensitizers according to guinea-pig or human data and with a LogP ≥3.5 included in the original LLNA validation, and the issue was never addressed specifically, requiring a closer look at the validity of this reference standard within this physicochemical range prior to assuming poor predictivity of the NAMs. With the recent extensive curation of robust reference datasets, including human data, there now exists an opportunity to better analyse the performance of the LLNA for lipophilic substances.

Given that the observation of reduced sensitivity at LogP≥ 3.5 was made for multiple alternative assays and defined approaches integrating them, there exists the theoretical possibility that, actually, the LLNA produces more false-positives in the physiochemical range of LogP≥ 3.5 rather than presuming that all *in vitro*/*in chemico* methods and NAMs, irrespective of their nature and solvent used (e.g. peptide reactivity, cell membrane surface receptor activation, cell media, DMSO, water) have the same predictive limitation. A higher number of false-positives for the LLNA test in the high LogP range would automatically lead to an apparent reduced sensitivity of all the alternative methods and DAs in which such methods are integrated when evaluating them against the LLNA as a reference. Thus, there is a need for scrutinizing LLNA reference data to conclude whether they are a correct reference when validating NAMs for lipohilic chemicals. The question is not: “Is the reference data (LLNA) wrong in general at LogP ≥3.5” but rather: “Is there an *increased rate* of LLNA false-positive for more lipohilic chemicals.” Here we analyse this question based on the curated OECD DASS reference database and based on other published datasets and show that indeed there is an increased false-discovery rate of the LLNA for lipophilic chemicals. We further propose a mechanistic explanation for this observation, whereby irritation triggered by lipophilic chemicals acts as a potential confounder.

## 2 Materials and methods

### 2.1 Short description of the OECD DASS reference DB

The DASS LLNA and human data sub-group (HDSG) database was constructed starting from a database collated previously by Cosmetics Europe (Hoffmann et al., 2018) and published jointly with the United States National Toxicology Program Interagency Center for the Evaluation of Alternative Toxicological Methods (NICEATM). The chemical set was expanded based on additional chemicals with LLNA and *in* *vitro* data as reported in Urbisch et al. (2015) and complemented with additional publicly available LLNA reference data. In parallel, all available data from human repeat insult patch tests (HRIPT) and human maximisation tests (HMT) were collected, including data provided by the Research Institute for Fragrance Materials (RIFM), to generate the HDSG dataset. The dataset was subject to a strict curation procedure which is described in detail for LLNA data in the Annex 3 (OECD, 2021b) and for human data in Annex 4 (OECD, 2021c) of the Supporting Document to OECD TG 497. Some of the most relevant criteria which explain the final composition of the DASS DB are listed below.

#### 2.1.1 LLNA

- In the LLNA reference database, study results were accepted as negative if for all test concentrations SI values were <3 and if the test substance was tested up to a highest concentration tested of at least 50% unless a justification was available that the tested concentration was the highest achievable (for technical or toxicological reasons).

#### 2.1.2 HDSG human data

- Only substances with data from HRIPT or HMT were included; clinical data and history of use data were excluded from the evidence, in contrast to the compilation by Basketter et al. (2014)
- Only substances that were tested at ≥25% and did not trigger sensitization were considered NC

#### 2.1.3 Basketter et al. human data

- All chemicals from the original Cosmetics Europe data collection had weight-of-evidence expert calls for their human sensitization potential that considered clinical data and history of use. No criteria were used to filter out chemicals from the Basketter *et al.* database; instead published Basketter et al. (2014) calls were added to the DASS database chemicals with agreed-upon LLNA classifications.

The DASS DB contains 168 chemicals with unambiguous LLNA hazard classifications agreed upon by the OECD DASS EG, from now on this will be referred to as DASS DB-168 (Table 1). These 168 chemicals correspond to 135 LLNA positive (sensitizers) (80%) and 33 LLNA negative (non-sensitizers) (20%). Of these 168 chemicals, only 56 have HDSG human reference classifications considered unambiguous by the EG, and these chemicals correspond to 47 human positives (84%) (also named human sensitizers throughout the text) and 9 human negatives (16%) (also named human non-sensitizers throughout the text).

**Table 1:** Composition of the DASS DB-168 in terms of LLNA and HDSG results, and their distribution based on LogP values.

Subset	Total	Positive	Negative
**LLNA**	168	135 (*80%*)^1)^	33 (*20%*)
**LogP≥3.5**	39 (23%)	33 (24%)	6 (18%)
**LogP<3.5**	129 (77%)	102 (76%)	27 (82%)

**HDSG**	56	47 (*84%*)	9 (*16%*)
**LogP≥3.5**	12 (21%)	6 (13%)	6 (66%)
**LogP<3.5**	44 (79%)	41 (87%)	3 (33%)

**Basketter**	96	68 (*71%*)	31 (*29%*)
**LogP≥3.5**	25 (26%)	15 (23%)	10 (32%)
**LogP<3.5**	71 (74%)	53 (77%)	18 (68%)

^1)^ Values in parenthesis correspond to the proportion that each value represents with respect to the total of that row or column. The percentages of the LLNA and HDSG rows (shown in italics) are with respect to the total of the row (e.g. for the LLNA Positives = 135 (80%), the percentage of 80% is with respect to LLNA Total, 135/168 = 80%), while the percentages of the LogP rows are with respect to the total of the column (e.g. for LLNA LogP**≥**3.5 Positives = 33 (24%), the percentage of 24% is with respect to the LLNA Positives, 33/135 = 24%).

Human evidence is available for more than these 56 chemicals: specifically, 96 chemicals out of the DASS DB-168 were assessed for their human skin sensitization potential in Basketter et al. (2014) (referred throughout the text as the Basketter database). This analysis took human evidence from different sources, namely clinical data, history of safe, widespread use, and HMT and HRIPT data into account. This expert judgment lacked detailed documentation on the sources of the clinical and use data and the way in which they were selected, evaluated and used in the overall assessment, and did not follow the strict rules applied by the DASS EG data review, but it has the advantage of presenting a much larger dataset. While the classifications provided in Basketter *et al.* were not considered to be sufficiently transparent by the OECD DASS EG, the Basketter database was extensively peer-reviewed and has been used multiple times as reference human data (Hoffmann et al., 2022; Kleinstreuer et al., 2018) and was part of the work that formed the basis of the US EPA science policy on accepting alternatives for skin sensitization (EPA, 2018). In addition, the classifications of the Basketter database are largely consistent with HDSG classifications for chemicals contained in both datasets (96%; N = 53 out of 55). The Basketter database has some valuable strengths such as a less biased dataset towards sensitizing substances (the Basketter dataset contains 71% of sensitizers while the HDSG contains 84% of sensitizers), a total of 96 chemicals with human data (almost twice the number of chemicals with HDSG data), and 28 human non-sensitizers (three times the number of human non-sensitizers in the HDSG). We agree that the Basketter database has not been subject to such strict criteria as the HDSG, nevertheless we consider that it provides valuable information, increases the number of chemicals with human data, and is another useful dataset to assess the hypothesis presented in this work without compromising its quality.

### 2.2 LogP data and classification

LogP values were all taken from the DASS database and include both measured and calculated values as indicated in the database.^1^ The distribution of substances of the DASS DB-168 with respect to their LogP values and skin sensitization potential (LLNA and HDSG) is shown in Table 1. The threshold of LogP≥3.5 had initially been defined by (Takenouchi et al., 2013) based on the empirical observation of 13 false-negative chemicals of the h-CLAT vs LLNA reference data in this physicochemical range in a database of 143 chemicals, and was included as a cutoff for reliable results in both TG 442E and TG 497 for DASS that use h-CLAT as an information source. This threshold has thus been used in this work to discriminate chemicals with high LogP (LogP≥3.5) and chemicals with low LogP (LogP<3.5).

The DASS DB-168 contains 39 chemicals with LogP≥3.5 (23% of the total), and 129 chemicals with LogP<3.5 (77%). The majority of the high LogP chemicals (N = 33) and low LogP chemicals (N = 102) are positive in the LLNA. Within the chemicals tested negative in the LLNA (N = 33), 6 (18%) correspond to chemicals with LogP≥3.5 and 27 (82%) to chemicals with LogP<3.5. Out of the 56 chemicals with HDSG data, 12 (21%) are chemicals with high LogP and 44 (79%) are low LogP. Within the HDSG chemicals classified as human sensitizers, 6 (13%) are high LogP chemicals and 41 (87%) are low LogP. Within the HDSG chemicals classified as human non-sensitiers, 6 (66%) are high LogP chemicals and 3 (33%) low LogP. A similar distribution to that of HDSG is observed for the Basketter database, 25 (26%) chemicals are high LogP chemicals and 71 (74%) low LogP. Within the chemicals classified as human sensitizers, 15 (23%) are high LogP and 53 (77%) low LogP. However, the distribution of the Basketter database for chemicals classified as non-sensitizers in humans is different from that of HDSG and more similar to the LLNA distribution, both in number of chemicals as well as proportion, as 10 (32%) chemicals are high LogP and 18 (68%) low LogP.

## 3 Results

### 3.1 Analysis of the DASS reference database: Predictivity of LLNA vs human data for chemicals below and above the LogP 3.5 threshold

#### 3.1.1 Predictivity of the LLNA vs HDSG data

In order to test whether the LLNA has an increased rate of false-positives compared to human data for substances with high LogP, the predictivity of LLNA with respect to HDSG calls in the DASS DB was analysed. Performance statistics were calculated for the overall dataset, and for the subsets of substances with LogP<3.5 and LogP≥3.5 (Table 2).

**Table 2:** Predictivity of the LLNA for DASS DB chemicals which have LLNA and HDSG data available (n = 56). Three subsets have been calculated: all (N = 56), LogP<3.5 (N = 44), and LogP≥3.5 (N = 12).

Set	FN	FP	TN	TP	N	Acc.	Bal-Acc	Sens.	Spec.	FDR 2)	Prev.
All	3	7	2	44	56	0.82	0.58	0.94	0.221)	0.14	0.8
LogP<3.5	3	2	1	38	44	0.89	0.63	0.93	0.33	0.05	0.93
LogP≥3.5	0	5	1	6	12	0.58	0.58	1	0.17	0.46	0.5

1 The specificity values are based on a relatively low number of chemicals compared to sensitivity values. Clopper Pearson 95% confidence intervals were thus calculated: The CI for the specificity of 0.22 is [0.02, 0.60].

2 False-discovery rate FDR indicates the proportion of false positive within the positive predictions (FDR=FP/(FP + TP)).

Table 2 shows that out of a total of 56 substances with LLNA and HDSG data, 10 have discordant LLNA and HDSG data (18%). Out of these 10 substances, 7 correspond to FPs and 3 to FNs. In terms of LogP<3.5 and LogP≥3.5 subsets, 5 of the discrepancies correspond to LogP<3.5 substances and the other 5 to LogP≥3.5 substances. While the number of substances with discordant results is the same for both subsets, their proportion is higher for the LogP≥3.5 subset, as this subset contains a total of 12 substances (5/12 = 42%) while the other contains a total of 44 substances (5/44 = 11%). This difference in proportion is clearly shown by the False Discovery Rate, which is of 0.46 for the LogP≥3.5 subset and 0.05 for the LogP<3.5 subset.

The table above shows that the LLNA is very good at predicting human sensitizers, since 44 out of the 47 human sensitizers are correctly predicted by the LLNA. This is reflected in a sensitivity of 0.94. This predictivity for human sensitizers seems not to be dependent on LogP as the sensitivity for the two sets, LogP<3.5 and LogP≥3.5, is 0.93 and 1.0, respectively. However, the LLNA is not good at predicting human non-sensitizers. Only 2 out of the 9 human non-sensitizers are correctly predicted by the LLNA. This is reflected in a specificity of 0.22. This value is highly uncertain due to low number of chemicals, but the 95% confidence interval indicates that specificity indeed is below 0.6. Specificity changes significantly between LogP<3.5, i.e. 0.33 (1 correctly predicted HDSG non-sensitizer out of 3) and LogP≥3.5, i.e. 0.17 (1 correctly predicted out of 6). These observations cannot be considered conclusive as it is difficult to establish a cause-effect relationship with this low number of substances.

##### 3.1.1.1 Interpretation of the results

The table above clearly shows poor performance of the LLNA when predicting HDSG non-sensitizers. The overall specificity of 0.22, although with a rather low number of chemicals (*i.e.* 2 correct predictions out of 9 HDSG non-sensitizers), is an indication that the LLNA is prone to provide FP results.

Analysing the data using the False Discovery Rate (FDR), which indicates the proportion of false positives within the positive predictions (FDR=FP/(FP + TP)), provides a revealing perspective. There are 40 chemicals in the DASS DB with LogP<3.5, positive LLNA results, and human data. Of these, only 2 correspond to HDSG non-sensitizers, thus 2FP of the LLNA with respect to HDSG. Therefore, the FDR for the LLNA in the range of low LogP substances is 2/40 (5%). There are 11 chemicals with LogP≥3.5, positive LLNA and human data. Of these, 5 correspond to HDSG non-sensitizers, thus the FDR of LLNA in the high LogP range is 5/11 (46%). These results suggest that the chances that positive results of LLNA tests for LogP ≥3.5 correspond to human non-sensitizers are much higher than for the positive results of LLNA tests for LogP< 3.5 chemicals. The results presented above clearly show higher FDR for LogP≥ 3.5 chemicals, however it is also clear that the low number of chemicals does not allow for deriving strong conclusions.

In order to shed light on the increased FDR rate of LLNA for high LogP substances, we further explored the Basketter et al. (2014) human data compilation, as it significantly enlarges the number of substances of the DASS DB-168 that have human data from 56 to 96.

#### 3.1.2 Predictivity of the LLNA vs Basketter et al. human data collection

Compared to the DASS reference database, the LLNA has clearly a higher predictivity for human data when evaluated with the Basketter *et al.* dataset (Bal-Acc = 0.58 for HDSG and 0.69 for Basketter), which also comprises a more balanced dataset with 68 sensitizers and 28 non-sensitizers (Table 3).

**Table 3:** Predictivity of the LLNA for chemicals of the DASS DB which have LLNA and human data from Basketter et al. (n = 96). Three subsets have been calculated: all (N = 96), LogP<3.5 (N = 71), and LogP≥3.5 (N = 25).

Set	FN	FP	TN	TP	N	Acc.	Bal-Acc	Sens.	Spec.	FDR	Prev.
All	1	17	11	67	96	0.81	0.69	0.99	0.391)	0.20	0.71
LogP<3.5	1	8	10	52	71	0.87	0.77	0.98	0.56	0.13	0.75
LogP≥3.5	0	9	1	15	25	0.64	0.55	1	0.10	0.38	0.60

1) The specificity values are based on a relatively low number of chemicals. Clopper Pearson 95% confidence intervals were thus calculated: The CI for the specificity of 0.39 is [0.21, 0.59].

Table 3 shows that out of a total of 96 substances, 18 have discordant LLNA and human data (Basketter et al., 2014). This corresponds to 19% of the substances, which is similar to the 18% of HDSG. Of these 18 substances, 17 correspond to FPs and only 1 to FNs. Nine of the discrepancies correspond to LogP<3.5 and the other 9 to LogP≥3.5 substances. While the number of substances with discordant results is the same for both subsets, their proportion is higher for the LogP≥3.5 subset, as this subset contains a total of 25 substances (9/25 = 36%) while the LogP<3.5 subset contains a total of 71 (9/71 = 13%). Despite the difference in absolute numbers, the percentage of discrepancies and their distribution within the subsets is almost identical to that of the LLNA-HDSG comparison. Observing the same trend with the larger number of substances in the LLNA-Basketter data adds weight to the hypothesis derived from the LLNA-HDSG comparison.

The excellent capacity of LLNA to predict human sensitizers detected in the LLNA-HDSG is also observed for the Basketter dataset, with a sensitivity of 0.99, independent of the LogP, (0.98 and 1.0 for LogP<3.5 and LogP≥3.5, respectively). The observation that the LLNA is not good at predicting human non-sensitizers is reproduced with the Basketter dataset as only 11 out of the 28 human non-sensitizers are correctly predicted by the LLNA. This results in a specificity of 0.39, which although almost doubling that of the LLNA-HDSG comparison, is still poor. Again, this value may be influenced by the low number of chemicals, but the 95% confidence interval upper limit is still only 0.59, which indicates high confidence in the low specificity of LLNA. Specificity also changes significantly for LogP<3.5 and LogP≥3.5 subsets in the Basketter dataset as it is 0.56 (10/18 non-sensitizers correctly predicted) and 0.10 (1/10 non-sensitizer correctly), respectively.

The Basketter LogP≥3.5 subgroup contains 4 additional human non-sensitizers than the HDSG data (N = 10 vs N = 6) and all 4 are incorrectly predicted by the LLNA. The predictivity of human non-sensitizers of the LogP≥3.5 subgroups for Basketter and HDSG are both poor, with 9/10 and 5/6 mispredicted, respectively.

##### 3.1.2.1 Interpretation of the results

The comparison of the DASS DB-168 substances with LLNA data and human data compiled by Basketter et al. (2014) shows a very similar picture to that provided by the LLNA-HDSG comparison. In both comparisons the LLNA provides discordant classifications in ∼20% of the cases, the proportion being higher for LogP≥3.5 substances (∼40%) than LogP<3.5 (∼10%). In both comparisons, the predictivity of the LLNA for human sensitizers are excellent, with sensitivity values ≥ 90% irrespective of the LogP value, and being close to 1.0 for the LLNA-Basketter comparison. The specificities of the two LogP subsets in the LLNA-Basketter comparison are significantly different, being 0.56 for LogP<3.5 and 0.1 for LogP≥3.5 substances. Therefore, the more comprehensive Basketter *et al.* data, although compiled according to less stringent criteria, confirms the trends observed in the LLNA-HDSG comparison.

Both datasets, LLNA-HDSG and LLNA-Basketter show an increased rate of FP vs human data in the LLNA-positives for LogP≥3.5 substances. In the LLNA-HDSG case, only 1 out of 6 human non-sensitizers is correctly predicted by the LLNA (specificity = 0.17), while for the LLNA-Basketter comparison, only 1 out of 10 human non-sensitizers is correctly predicted by the LLNA (specificity = 0.1) in this physicochemical range.

The use of the human data compiled by Basketter et al. (2014) has shed light on the predictivity of the LLNA vs human. The LLNA-HDSG LogP<3.5 subset had a high FP rate (2 FPs out of 3 human non-sensitizers, *i.e.* specificity = 0.33) possibly due to the small number of substances present in the subset. In fact, the data compiled by Basketter et al. shows that this was probably the case, as it contains 8 FP out of 18 human non-sensitizers, *i.e.* specificity = 0.56 which is closer to the commonly reported values (Haneke et al., 2001; Hoffmann et al., 2018, 2022) for the specificity of LLNA vs human data. The prevalence of sensitizers/non-sensitizers is more balanced in the LLNA-Basketter data compared to LLNA-HDSG: 0.73 instead of 0.93, respectively. The LLNA-HDSG LogP≥3.5 subset showed an even higher FP rate with 5 FP out of 6 human non-sensitizers. In this case, however, the Basketter *et al.* data, has not changed the observation of a higher FP rate, rather, it has reinforced it, as the 4 extra human non-sensitizers found in the Basketter *et al.* data are all FP.

### 3.2 Analysis of the DASS reference database: weight of evidence analysis for individual chemicals to identify and understand potential false-positive chemicals in the LLNA

Next to HRIPT and HMT data, other evidence may be consulted on whether a chemical is a human non-sensitizer (use data, clinical patch test data) as done by Basketter et al. (2014). These data can be used in a weight-of-evidence to conclude whether a chemical is truly a skin sensitizer, although they do not meet the criteria set for inclusion in the DASS HDSG reference data. We reviewed all chemicals positive in the LLNA in the DASS-DB, and a detailed documentation on a chemical-by-chemical level is provided here for chemicals for which we consider sufficient evidence is available to rate them as human non-sensitizers (Supplementary Table S1). This documentation is more detailed than that provided by Basketter et al. (2014). Based on this analysis, the eight lipophilic chemicals in Table S1 (Hexyl salicylate, Benzyl benzoate, Citronellol, (R)-(+)-Limonene, Tocopherol, Isopropyl myristate, iso-Methylionone and OTNE) are not relevant human sensitizers despite a positive LLNA result, and could thus be regarded as LLNA false-positives. Given that the DASS database contains 33 LLNA positives with a LogP ≥3.5, 8 *bona fide* false-positives vs human data identified by this WoE analysis in Table S1 indicates a FDR rate of at least 24% vs human data.

Among the 102 chemicals with a LogP <3.5 and positive LLNA classifications, we identified only four potential false-positives in a WoE analysis (Table S2). This indicates a FDR of at least 4% *bona fide* false-positives in the DASS-DB for chemicals with LogP <3.5. This analysis cannot be considered comprehensive; there may be other chemicals in the database, in both physicochemical ranges, for which the LLNA might be false-positives, but this cannot be determined due to a lack of human data, and the chemicals in Tables S1 and S2 are just those for which we consider sufficient evidence is available. Therefore, the exact difference in LLNA specificity and LLNA FDR between the two physicochemical ranges cannot be known for the full DASS database, but this additional analysis indicates that based on evidence collected for single chemicals, an increased FDR at LogP ≥3.5 appears to be confirmed when looking at the full DASS database.

### 3.3 Analysis of evidence outside of the DASS reference database: LLNA data compared to other references (Guinea pig and *in silico*)

To understand whether the increased FDR of the LLNA at LogP ≥3.5 when compared to human data is a new finding, it is instructive to also look at data beyond the curated DASS DB and to consult the peer-reviewed literature. The primary reference data when validating the LLNA, next to human data, were guinea pig data (GPMT and Buehler test) (Dean et al., 2001; Haneke et al., 2001; Sailstad et al., 2001). The advantage of the guinea pig assay is that it truly measures the elicitation phase of skin sensitization. False-positive LLNA results vs the guinea pig data have been repeatedly discussed in the literature, both during the validation process but also after the LLNA became an OECD guideline. Here we highlight the results from two key studies. (i) Ball et al. (2011) reported, next to sodium dodecyl sulphate (SDS), five surfactants that are FP in the LLNA when assessed vs guinea pig maximisation test; four of those have a LogP ≥3.5. (ii) Kreiling et al., 2008, 2017 reported 6 ‘unsaturated compounds’ as FP in the LLNA when assessed vs guinea pig maximisation test. Four of those have a LogP ≥3.5 and include simple fatty acids and the most prominent hydrocarbon endogenous to the human skin, namely squalene. Details of the false-positives with a LogP≥ 3.5 as compared to guinea-pig data in these two detailed studies are shown in Table 4. These two studies indicate that at a high LogP, a number of chemicals return false-positive results in the LLNA when compared to guinea pig data.

**Table 4:** Lipophilic chemicals which were rated as false-positive vs guinea pig in the studies by Kreiling et al. and Ball *et al.*

CAS	Structure	Name	cLogP Chem-Draw	LLNA Result (EC3%)	Guinea pig result
**Study of Kreiling *et al.***					
112-80-1		Oleic acid	7.8	10.4	negative
60-33-3		Linoleic acid	7.3	14	negative/ambiguous
463-40-1		Linolenic acid	6.8	10	negative
111-02-4		Squalene	12.9	<10%	negative
**Study of Ball *et al.***					
n.a.		Hexadecan-1-ol, Ethoxylated (2 EO)	7	1.2	negative (0/20)
n.a.		Decylphenolpolyethylene glycol ether	4.8	2.0	negative (0/20)
n.a.		Hexaethylene glycol monododecyl ether	4.2	7.4	negative (0/20)
n.a.		Tetraethylene glycol monotetradecyl ether	5.6	2.8	negative (0/20)

In a study on the predictivity of an *in silico* model to predict reactive skin sensitizers vs LLNA data (Patlewicz et al., 2007), a low sensitivity (56%) was reported for the *in silico* model with an external test set. While the true human sensitization status of those chemicals is not known, it is interesting to note that among the seven chemicals (7 of 16 tested) considered false-negative vs LLNA in the *in silico* model, there are four chemicals with no apparent structural alert for skin sensitization, including a simple alkane. All four of these chemicals have a LogP ≥3.5 (Table 5). Based on the absence of structural alerts, these chemicals, according to our assessment, could be putative false-positives in the LLNA rather than false-negatives in the *in silico* model.

**Table 5:** Lipophilic chemicals positive in the LLNA and miss-predicted by TIMES SS as non-sensitizers in (Patlewicz et al., 2007).

CAS		cLogP	LLNA	TIMES SS a	Notes
140-26-1		3.84	1	0	No indication that aliphatic esters are sensitizers, both cleavage products are non-sensitizers, considered non-sensitizer (Api et al., 2018) based on read-across to CAS 140-26-1 which is non-sensitizing in GP
2136-71-2		6.4	1	0	Ethoxylated surfactant, no sensitization evidence for this widely used surfactant category (Ball et al., 2011)
629-50-5		6.3	1	0	No evidence for sensitization for alkanes
613-29-6		4.7	1	0	No structural alert, eye and skin irritant according to ECHA

a TIMES SS result at the time of the publication.

The studies summarized in Table 4, Table 5 indicate that independent studies found discordant positive results in the LLNA vs guinea pig data or reactivity alerts, and these putative false-positives cluster in the range of LogP≥3.5, thus giving supporting *evidence* to the analysis on the DASS reference database conducted above. These chemicals include a number of surfactants, but also other highly lipophilic chemicals which are clearly not surfactants such as squalene or tridecane.

### 3.4 Analysis of the LLNA validation set

Finally, a key question remains: if such a lower specificity of the LLNA at LogP ≥3.5 exists, why had it not been recognized earlier? To answer this question, we investigated the list of chemicals analysed when validating the LLNA (Haneke et al., 2001). We could retrieve the chemical structure and hence could calculate a cLogP for 194 chemicals from the chemicals analysed by ICCVAM. 129 of those have a LogP <3.5. Out of these, 96 have guinea pig data with 27 being negative in guinea pig tests. Of the guinea pig negatives, 24 are also negative in the LLNA, showing a high specificity of the LLNA in the range of LogP <3.5 (89%). The dataset contains many chemicals with a LogP ≥3.5 (N = 63), however guinea pig data were available for only 20 of those, including only four negative chemicals. Two of those four are positive in the LLNA, which would give a specificity of 50%, but given this low sample number it is not surprising that this issue was never of concern during validation and upon implementation of OECD TG429. Also, it has to be re-iterated that during validation most negatives in the guinea pig test were only tested in the LLNA up to 25% (Kolle et al., 2020), while the DASS curation criterion required a test concentration of at least 50% to accept a negative LLNA study and OECD TG429 even requires testing up to 100% if no apparent irritation is observed. The higher test concentration introduced after the validation (Kolle et al., 2020) may have contributed to a higher number of false positives and a decreased specificity.

### 3.5 A potential mechanistic explanation for a higher FDR of the LLNA for lipohilic chemicals

The analyses of different (and partly overlapping) sets of reference data consistently indicate an increased FDR at high LogP for the LLNA; however, this is purely observational and often based on a small sample number. Thus, understanding a potential mechanism underlying this observation would add to the weight this observation may have when assessing relevance of reference data. The key confounder for potential false-positives in the LLNA discussed in the literature is skin irritation, leading to non-specific cell proliferation in the lymph node. Thus, based on skin irritation as a confounder, the following sequence of events can be postulated leading to a false-positive in the LLNA for a high LogP molecule.

1. High LogP molecules in general have an increased cytotoxicity. Correlation between (cyto)toxicity and LogP is very well described in the literature on ecotoxicology, see e.g. (Tebby et al., 2011).
2. Cytotoxic molecules tend to have increased skin irritation potential. Accordingly, all OECD accepted 3D skin- and eye- irritation methods measure cytotoxicity to the tissue model as the key read-out to identify irritants (Kandarova et al., 2005; Spielmann et al., 2007).
3. Skin irritation by cytotoxic molecules involves release of interleukin-1α from damaged skin cells. Thus, IL-1α release is an add-on endpoint in skin irritation tests on 3D models (Cotovio et al., 2005; Spielmann et al., 2007)), but also other cytokines such as TNF-alpha (Kock et al., 1990) or IL-6 (Kirnbauer et al., 1991) are released by damaged keratinocytes.
4. IL-1α (and maybe other cytokines) can trigger irritant-induced dendritic cell emigration from the skin to the lymph nodes similarly as IL-1β can do for allergens (Cumberbatch et al., 2002) and IL-1α can induce lymph node cell proliferation.

Thus, lipophilic chemicals, especially when tested at high concentration, may (partly) damage cells on the skin surface of the mouse ear. This may lead to a local release of cytokines such as, *inter alia,* IL-1α. Released cytokines will trigger local inflammation and emigration of activated dendritic cells from the skin which then may induce non-specific cell proliferation in the lymph nodes.

To name an example for a specific chemical class, the ethoxylated surfactants from the Ball *et al.* publication (Ball et al., 2011), triggered dramatic levels of IL-1α release in the EpiSkin® model even when tested in dilutions and already at concentrations which did not yet lead to a full cytotoxicity of the integral tissue model. They were strongly (false-)positive in the LLNA (Ball et al., 2011).

This sequence of events, which are individually well established but have not been proven *in vivo* in the LLNA situation, provide a biologically plausible explanation of why high LogP molecules might trigger by their cytotoxic/irritant nature IL-1α/cytokine-induced lymph node cell proliferation. As the LLNA is based on general cell proliferation in the lymph nodes, the LLNA may, therefore, not perfectly discriminate some lipophilic cytotoxic molecules from sensitizers when tested at high doses. In this respect, it is important to note that many of the potential LLNA FP reported here were positive only at relatively high test concentrations.

## 4 Discussion

In summary, different lines of evidence summarized above indicate that indeed the specificity of the LLNA for high LogP compounds tends to be lower and the FDR higher. The analysis presented here does not indicate that the LLNA in general is wrong at higher LogP and that all positive LLNA results in this physicochemical range are questionable. Rather, different lines of evidence indicate that the FDR of the LLNA is higher for lipophilic chemicals, and this conclusion is made based on different (partly overlapping) datasets: (i) LLNA vs HDSG data (N = 56) show that the FDR of LLNA in the LogP<3.5 range is 0.05 (2/40) while for LogP≥3.5 range it is 0.46 (5/11); (ii) LLNA vs Basketter human data (N = 96) show the FDR in the range LogP<3.5 is 0.13 (8/60) while for the LogP≥3.5 range is 0.38 (9/24); (iii) there is strong evidence that at least 8/33 LLNA positive chemicals in the DASS database with LogP ≥3.5 are not relevant human sensitizers based on HDSG analysis and/or WoE, which translates into a FDR of the LLNA of ≥0.24 in the full database at LogP ≥3.5; (iv) the studies of Ball *et al.* and Kreiling *et al.*, which reported 11 FP of the LLNA with respect to GPMT data, include 8/11 chemicals with LogP≥3.5; and (v) out of the 7 lipophilic chemicals from Patlewicz *et al.* that were positive in the LLNA but had negative *in silico* predictions, 57% (4/7) had no structural alerts for sensitization, had a cLogP >3.5 and belong to widely used chemical categories with no evidence of sensitization.

A higher FDR of LLNA in the LogP≥ 3.5 range logically leads to a lower sensitivity of methods that are evaluated solely against the LLNA in this LogP range. For instance, if we carry out the fictitious exercise of determining how good the HDSG call would be at predicting skin sensitization using LLNA as the sole reference, we would find out that the sensitivity of HDSG in predicting skin sensitization would be 0.95 for LogP<3.5 chemicals and 0.55 for LogP≥ 3.5 chemicals. In such a thought experiment, this would lead to the conclusion that human data cannot be applied to chemicals with LogP≥ 3.5 due to decreased sensitivity in that range, while actually it is human risk we had wanted to predict. A method that would be as good as the HDSG call at predicting skin sensitization (i.e. a method perfectly mimicking the human situation reflected by this database), would have a sensitivity of only 0.55 for the subset of LogP≥ 3.5 when evaluated against the LLNA. Since the FDR for higher LogP chemicals is roughly 20% higher than that of the low LogP, for any new method or DA evaluated against the full DASS reference database we would expect a reduced sensitivity vs LLNA in the range of high LogP, if the approach is in fact predictive for human data. Interestingly, to name an example, the ‘2 out of 3’ DA indeed predicted Hexyl salicylate, Tocopherol, Isopropyl myristate, iso-Methylionone and OTNE as negative, *i.e.* five of the eight chemicals for which the WoE/HDSG data indicated no relevant human sensitization potential, while (R)-(+)-Limonene and Benzyl benzoate were in the borderline range.

Thus, this enhanced FDR of the LLNA for chemicals above LogP of 3.5 is a likely and at least partial explanation for the observed apparent drop in sensitivity of ca. 10–15% calculated for the DASS in this physicochemical range, and for the reduced sensitivity reported for the individual assays. An alternative explanation for the concomitant reduced sensitivity for all three NAMs brought forward is a potentially limited exposure by poorly soluble chemicals when tested in the aqueous (KeratinoSens™, h-CLAT) or partly aqueous (DPRA) incubation media. Indeed, limited solubility is widely perceived as an intrinsic limitation for *in vitro* testing. Nevertheless, a more in-depth evaluation of the data generated with the cell-based assays shows that the vast majority of the FN vs LLNA with a LogP ≥3.5, while not inducing the markers for positivity (luciferase induction in KeratinoSens or CD86/CD54 expression in h-CLAT), still lead to cytotoxicity in KeratinoSens™ and/or h-CLAT tests. Thus, for the h-CLAT 10/12 (83%) and for KeratinoSens™ 13/13 (100%) of the chemicals that were FN vs LLNA with a LogP ≥3.5 caused at least 25% (h-CLAT) or 50% (KeratinoSens) cytotoxicity in the tested concentration range. This observation proves that at the tested exposure concentrations, these chemicals did provide sufficient cell exposure. Furthermore, for the DPRA, a recent study (Yamamoto et al., 2019) investigated which of the 82 chemicals initially tested by Gerberick et al. (2007) to assess DPRA predictivity lead to visible precipitation under the DPRA test conditions. Precipitation would indicate that the solution is oversaturated and that the dissolved exposure concentration is below the nominal concentration. Analysing the data in that study, the predictivity of the DPRA as stand-alone method is still at 80% (20/25) for those partly dissolved chemicals. This indicates that the reaction can proceed with the amount of the test chemical being in solution, and additional test chemical may get dissolved as the dissolved test chemical has reacted, in case the reaction rate is slower than the dissolution rate. Taken together, these observations indicate that a limited exposure for more hydrophobic chemicals due to insolubility is not a general reason for not detecting the biological activity (in cell-based *in vitro* assays) or reactivity (in *in chemico*) assays.

It may be somewhat surprising that with the most rigorously curated LLNA database ever available (OECD, 2021b) - which is the result of a detailed evaluation and agreed-upon LLNA classifications within the OECD Defined Approach for Skin Sensitization Expert Group in the process of developing OECD GL497 -, such limitations become more apparent than ever before, but data curation must not be confounded with data relevance, as the curation effort indeed led to some surprises: (i) Of the five negative reference chemicals previously included as LLNA performance standards (OECD TG429), one (salicylic acid) is rated positive by the DASS EG data review, for two (chlorobenzene and methyl salicylate) no conclusive LLNA call could be made based on the review criteria, for one (lactic acid) a negative attribution could only be made by an expert assessment, and ultimately only one chemical (isopropanol) was considered to be clearly negative in the assessment. (ii) DMSO as a guideline-recommended LLNA vehicle is positive in the LLNA according to the DASS EG data review criteria. These observations, namely that negative standards and recommended solvents do not give the correct result anymore further indicate that the specificity of the LLNA as evaluated based on the DASS reference database may be different from that calculated during the validation of the LLNA. This may be partly linked to the fact that the evaluation criteria applied today are largely based on a required higher test dose in OECD TG429 as compared to the typical maximal test concentrations used during the LLNA validation (Kolle et al., 2020).

## 5 Conclusions

Currently, a limitation of the h-CLAT for accepting negative results for chemicals at LogP ≥3.5 forms part of OECD TG 442E. This then affects negative calls made with a DASS using h-CLAT as an information source (TG 497). The analysis presented here indicates that such cases should not be assumed unreliable, but rather should be evaluated based on a weight-of-evidence approach, especially also taking structural alerts into account. For chemicals for which adequate exposure of the cells is proven by cytotoxicity and which lack a clear structural alert for reactivity, negative calls should be accepted based on a WoE, rather than rejected and proceeding to LLNA testing. Rejecting *in vitro* data for high LogP substances is not an appropriate solution because it will likely lead to conducting LLNA studies in exactly the physicochemical range at which LLNA predictivity is lower, as demonstrated in this study.

The analyses presented here may encourage a removal of the limitation for lipohilic molecules contained in TG 442E for the h-CLAT, upon the next revision of the guideline text. Importantly, this potential data bias also needs to be taken into account when developing new NAMs/DASS to specifically predict the LLNA outcome. This is particularly important for assays based on a high number of input variables such as rule-based *in silico* models, *in silico* models based on a large number of chemical descriptors or tests including genomic data. Such approaches with a large number of input variables have an intrinsic risk for data overfitting, and training new models using the LLNA database may then replicate these observed weaknesses of the animal test.

## Funding

Givaudan Schweiz AG has paid the salary of Andreas Natsch, National Toxicology Program Interagency Center for the Evaluation of Alternative Toxicological Methods has paid the salary of Nicole Kleinstreuer, and European Commission, Joint Research Centre has paid the salary of David Asturiol and the open access fee. None of the sponsors above have been involved in the study design; in the collection, analysis and interpretation of data; in the writing of the report; or in the decision to submit the article for publication.

## CRediT authorship contribution statement

**Andreas Natsch:** Conceptualization, Formal analysis, Investigation, Data curation, Validation, Writing – original draft, Writing – review & editing, Project administration. **Nicole Kleinstreuer:** Conceptualization, Data curation, Validation, Writing – review & editing. **David Asturiol:** Conceptualization, Validation, Formal analysis, Investigation, Data curation, Writing – original draft, Writing – review & editing.

## Declaration of competing interest

The authors declare that they have no known competing financial interests or personal relationships that could have appeared to influence the work reported in this paper.

## Data Availability

The data used is referenced in the manuscript as it is publicly available, and extra data is included in the supporting information

## Footnotes

1 Kathon CG is a mixture of Methylchloroisothiazolinone (MCI) and Methylisothiazolinone (MI) with a defined proportion of about 76% MCI and 24% MI and it did not have an assigned LogP value in the DASS DB-168. Both components of the mixture have calculated LogP<3.5 and according to the Kathon CG data safety sheet, the measured LogP is 0.401. Thus, in our analysis we considered this mixture to belong to the group of substances of LogP<3.5.
