# Supplementary material for: Reduced specificity for the local lymph node assay for lipophilic chemicals: Implications for the validation of new approach methods for skin sensitization
Source: Regul Toxicol Pharmacol. 2023 Feb;138:105333. doi: 10.1016/j.yrtph.2023.105333 (PMC9941753; doi:10.1016/j.yrtph.2023.105333)
Supplement: Multimedia component 1 [file mmc1.docx]

**Table S1. Lipophilic chemicals in the DASS database which are rated as sensitizers by LLNA, but which are non-sensitizers in humans based on the DASS analysis and/or a WoE analysis**

| Chemical | CASRN | LLNA MLLP | HU GHS | LogP | Details and Reference |
| --- | --- | --- | --- | --- | --- |
| Hexyl salicylate   | 6259-76-3 | 14.09 | NC | 3.18 (5.5 exp. REACH) | Considered human non-sensitizer by HDSG on available predictive tests, no structural alert |
| Benzyl benzoate   | 120-51-4 | 17 | NC | 3.97 | Considered human non-sensitizer by HDSG based on available predictive tests; very low SEQ^[[1]](#footnote-1)^ in Schnuch *et al*.(Schnuch et al., 2015) (0; based on 1 positives in 2003 tests (Schnuch et al., 2007)) indicates very low frequency of clinical reaction compared to volume of use |
| Citronellol   | 106-22-9 | 43.5 | NC | 3.91* | Considered human non-sensitizer by HDSG. Has been tested at 25% in 101 panellists with no positive reactions (see human subgroup report, RIFM08). SEQ 0.12/0.1 in Schnuch *et al.*(Schnuch et al., 2015) indicates very low frequency compared to use volume. (As for limonene and linalool, artificially oxidized samples are positive (Rudback et al., 2014)) |
| (R)-(+)-Limonene   | 5989-27-5 | 52.5 | NC/1B | 4.51 | For Commercial Limonene very rare cases of contact allergy are reported, despite very widespread use. The SCCS opinion on fragrance materials (2012) lists patch test data on 5500 subjects with only ca. 15 reported cases globally over many years, despite very widespread use of limonene (present in majority of cosmetic products investigated, thus almost daily contact for the whole population). Schnuch et al. (Schnuch et al., 2015) indicated SEQ of 0.07/0.08, *i.e.* at the very low end of the scale. Very high exposure to almost pure limonene occurs regularly upon manually peeling of citrus fruits widely in the population and there are very rare / no reports on incompatibility of skin with citrus fruit handling by consumers (isolated case for citrus picker where high exposure is continuous over years mentioned in SCCS review). Next to the study mention in the database (8% on n=25 with no effects), there is a Kligman Maximisation test in RIFM database at 20% on n=25 with no effects.  Based on both the clinical data (in relation to use) and the maximisation data, Limonene non-oxidized should be rated NC and not confused with oxidized limonene (oxidized by open stirring for weeks to months)(Karlberg et al., 1992). Also, the SCCS opinion states: “The allergenicity of limonene is closely related to oxidation”.) Oxidized limonene also generates positive in vitro data. |
| Tocopherol   | 59-02-9 | 7.4 | NA | 9.4 | Vitamin E, endogenous antioxidant in human skin, very widely used in topical products with very rare cases of sensitization compared to frequency of use. Sensitization to endogenous compounds is generally rare (Kosari et al., 2010). Main reactions reported for esters of Tocopherol, but synthetic tocopheryl esters do have an additional alert, as phenol esters they are good acyl transfer agents. |
| Isopropyl myristate   | 110-27-0 | 44 | NC/1B | 6.9 | Very widely used solvent in perfumery and cosmetics, very rare positive clinical reactions, no structural alert. Negative at 20% in human HRIPT (25 subjects only), Simple alkyl esters lack reactivity and acyl-transfer potential, considered ‘extremely weak or non-sensitizer’ based on extended patch-testing (16 reaction in 12’600 patients; less frequent than *e.g.* propylene glycol)(Uter et al., 2004). |
| iso-Methylionone   | 127-51-5 | 21.8 | NC | 4.38 | Negative by HDSG analysis. Has been tested at 60% in 106 and 23 panellists with no positive reactions (see human subgroup report, RIFM08). Very low frequency of positive reactions (n=1 of 2004; (Schnuch et al., 2007)in clinic despite low use leading to high SEQ 0.23/0.08 Schnuch *et al.*(Schnuch et al., 2015)).  Has a Michael acceptor alert, but was shown to be completely shielded leading to not even traces of peptide adducts (Natsch and Emter, 2017) |
| OTNE   | 54464-57-2 | 14.2 | NC | 5 | Considered human non-sensitizer by the assessment of the HDSG. Has been tested at 40% in 101 and 22.5% in 53 panellists with no positive reactions (see human subgroup report, RIFM08). no structural alert |

* LogP from DASS database; ECHA reports cLogP of 3.55 and experimental LogP of 3.41, thus this chemical is borderline for the 3.5 threshold.

**Table S2. Chemicals with a LogP < 3.5 which are false-positive in the LLNA based on HDSG criteria and/or WoE**

| Chemical | CASRN | LLNA MLLP | HU GHS | LogP | Details and Reference |
| --- | --- | --- | --- | --- | --- |
| Salicylic acid   | 69-72-7 | 12.2 | NC/1B | 2.26 | Non-sensitizer in humans despite widespread topical use. Negative in human predictive test at 20%, see HDSG report. SCCS made a WoE assessment and concluded that salicylic acid is a non-sensitizer <https://op.europa.eu/en/publication-detail/-/publication/75be19bf-5390-11ea-aece-01aa75ed71a1>  Salicylic acid is very widely used up to 2% in leave-on cosmetics as active ingredient, but allergic reactions are not reported. Negative in Buehler test with 25% induction concentration. |
| Sodium lauryl sulphate   | 151-21-3 | 3.7 | NC | 1.6 | SDS is widely documented as false-positive in the LLNA(Loveless et al., 1996). Negative by HDSG. |
| Linalool   | 78-70-6 | 35.5 | NC/1B | 2.97 | SEQ 0.08/0.1 in Schnuch (Schnuch et al., 2015) indicates very low frequency compared to volume despite very high patch test concentration (10%). Linalool has an HRIPT NOEL of 13793 μg/cm^2^ (Basketter et al, 2014; Gerberick et al, 2001) and HMT NOEL of 55176 μg /cm^2^ (Greif, 1967) with no cases of sensitization reported in both tests conducted at high concentration, thus it is close from being labelled as a human NC by HDSG criteria (20% top test concentration as compared to 25% set as threshold).  The SCCNFP opinion on fragrance materials (2012) lists patch test data on 5423 subjects with only 18 reported cases globally (0.3%), despite widespread use of linalool (present in ≥ 95% of cosmetic products investigated). SCCNFP opinion lists Linalool as a well-established contact allergen in humans, however this assessment is largely based on data on Linalool put under forced oxidation for several months (Brared Christensson et al., 2012), which is not relevant for the regulatory assessment of the parent compound and also appears not to represent commercial use(Kern et al., 2014). For Linalool there is often a confusion made between artificially oxidized Linalool (open stirring for months (Sköld et al., 2004)) and the peroxide formed and normal, commercial Linalool which we assess here. Oxidized Linalool is also positive in *in vitro* assays. |
| DMSO **** | 67-68-5 | 72 | NC | -1.35 | Considered human non-sensitizer by the HDSG on available predictive tests; (Marren, 2011) |

**References**

Brared Christensson, J., Andersen, K. E., Bruze, M., Johansen, J. D., Garcia-Bravo, B., Giménez Arnau, A., Goh, C. L., Nixon, R., White, I. R., 2012. Air-oxidized linalool-a frequent cause of fragrance contact allergy. Contact Dermatitis. 67**,** 247-259. 10.1111/j.1600-0536.2012.02134.x

Karlberg, A. T., Magnusson, K., Nilsson, U., 1992. Air oxidation of d-limonene (the citrus solvent) creates potent allergens. Contact Dermatitis. 26**,** 332-40.

Kern, S., Dkhil, H., Hendarsa, P., Ellis, G., Natsch, A., 2014. Detection of potentially skin sensitizing hydroperoxides of linalool in fragranced products. Anal Bioanal Chem. 406**,** 6165-78. 10.1007/s00216-014-8066-3

Kosari, P., Alikhan, A., Sockolov, M., Feldman, S. R., 2010. Vitamin E and allergic contact dermatitis. Dermatitis. 21**,** 148-53.

Loveless, S. E., Ladics, G. S., Gerberick, G. F., Ryan, C. A., Basketter, D. A., Scholes, E. W., House, R. V., Hilton, J., Dearman, R. J., Kimber, I., 1996. Further evaluation of the local lymph node assay in the final phase of an international collaborative trial. Toxicology. 108**,** 141-52.

Marren, K., 2011. Dimethyl sulfoxide: an effective penetration enhancer for topical administration of NSAIDs. Phys Sportsmed. 39**,** 75-82. 10.3810/psm.2011.09.1923

Natsch, A., Emter, R., 2017. Reaction Chemistry to Characterize the Molecular Initiating Event in Skin Sensitization: A Journey to Be Continued. Chem. Res. Toxicol. 30**,** 315-331. 10.1021/acs.chemrestox.6b00365

Rudback, J., Hagvall, L., Borje, A., Nilsson, U., Karlberg, A. T., 2014. Characterization of skin sensitizers from autoxidized citronellol - impact of the terpene structure on the autoxidation process. Contact Dermatitis. 70**,** 329-339. 10.1111/cod.12234

Schnuch, A., Uter, W., Geier, J., Lessmann, H., Frosch, P. J., 2007. Sensitization to 26 fragrances to be labelled according to current European regulation. Results of the IVDK and review of the literature. Contact Dermatitis. 57**,** 1-10. 10.1111/j.1600-0536.2007.01088.x

Schnuch, A., Uter, W., Lessmann, H., Geier, J., 2015. Risk of sensitization to fragrances estimated on the basis of patch test data and exposure, according to volume used and a sample of 5451 cosmetic products. Flavour Frag. J. 30**,** 208-217. 10.1002/ffj.3241

Sköld, M., Börje, A., Harambasic, E., Karlberg, A. T., 2004. Contact allergens formed on air exposure of linalool. Identification and quantification of primary and secondary oxidation products and the effect on skin sensitization. Chem Res Toxicol. 17**,** 1697-1705.

Uter, W., Schnuch, A., Geier, J., Lessmann, H., 2004. Isopropyl myristate recommended for aimed rather than routine patch testing. Contact Dermatitis. 50**,** 242-4. 10.1111/j.0105-1873.2004.00352.x

1. Schnuch et al. calculated the incidence of positive reactions relative to use (both expressed as industrial use volume and frequency of labeling on consumer producs) as SEQ (“Sensitization Exposure Quotient”). Data from IVDK are used, which is a network of 62 dermatological centers, contributing their data to a very well curated central data repository. [↑](#footnote-ref-1)
